# Increases in Endogenous or Exogenous Progestins Promote Virus-Target Cell Interactions within the Non-human Primate Female Reproductive Tract

**DOI:** 10.1371/journal.ppat.1005885

**Published:** 2016-09-22

**Authors:** Ann M. Carias, Shannon A. Allen, Angela J. Fought, Katarina Kotnik Halavaty, Meegan R. Anderson, Maria L. Jimenez, Michael D. McRaven, Casey J. Gioia, Tara R. Henning, Ellen N. Kersh, James M. Smith, Lara E. Pereira, Katherine Butler, S. Janet M. McNicholl, R. Michael Hendry, Patrick F. Kiser, Ronald S. Veazey, Thomas J. Hope

**Affiliations:** 1 Department of Cell and Molecular Biology, Feinberg School of Medicine, Northwestern University, Chicago, Illinois, United States of America; 2 Department of Preventive Medicine, Feinberg School of Medicine, Northwestern University, Chicago, Illinois, United States of America; 3 Division of HIV/AIDS Prevention, Centers for Disease Control and Prevention, Atlanta, Georgia, United States of America; 4 Tulane National Primate Research Center, Tulane University School of Medicine, Covington, Louisiana, United States of America; Emory University, UNITED STATES

## Abstract

Currently, there are mounting data suggesting that HIV-1 acquisition in women can be affected by the use of certain hormonal contraceptives. However, in non-human primate models, endogenous or exogenous progestin-dominant states are shown to increase acquisition. To gain mechanistic insights into this increased acquisition, we studied how mucosal barrier function and CD4+ T-cell and CD68+ macrophage density and localization changed in the presence of natural progestins or after injection with high-dose DMPA. The presence of natural or injected progestins increased virus penetration of the columnar epithelium and the infiltration of susceptible cells into a thinned squamous epithelium of the vaginal vault, increasing the likelihood of potential virus interactions with target cells. These data suggest that increasing either endogenous or exogenous progestin can alter female reproductive tract barrier properties and provide plausible mechanisms for increased HIV-1 acquisition risk in the presence of increased progestin levels.

## Author Summary

Sexual transmission accounts for over 80% of all HIV-1 infections, with half of new infections occurring in women. Epidemiological studies suggest that certain hormonal contraceptives may be associated with increased HIV-1 acquisition. A hormonal influence of vaginal HIV acquisition is supported by studies utilizing various non-human primate models, which reveal that susceptibility to vaginal transmission is affected by the menstrual cycle or exogenous hormonal treatment. However, the mechanism of increased susceptibility remains unknown. Here, utilizing a variety of techniques and non-human primates, we illustrate that progestin-based contraceptives, such as Depo-Provera, and the natural menses luteal phase have a very similar impact in these models systems. Both increase virus influx into the endocervix and stimulate target cell infiltration of the squamous epithelium. The shedding of the superficial dead layers of the squamous epithelium in the progestin dominant state put the tissue resident cells in close proximity with virus in the lumen increasing the possibility of interactions and infection. A better understanding of vaginal SIV/SHIV acquisition in these widely utilized models is essential to interpret the results of ongoing clinical trials of HIV-1 acquisition during hormonal contraceptives use. An increased understanding of the mechanisms of HIV acquisition should contribute to vaccine development.

## Introduction

Globally, male-to-female mucosal transmission accounts for the majority of recent HIV-1 infections (60%) [1]. Numerous factors may increase HIV-1 acquisition risk in women, including stage of HIV-1 infection and viral load of the partner, co-infections with sexually transmitted infections, cervical ectopy, and age [2,3]. A consensus is now building that hormonal fluctuations and changes in the mucosal barriers of the female reproductive tract (FRT) are significant risk factors for HIV-1 acquisition. [4–11]. The underlying mechanisms behind increased acquisition remains to be elucidated.

During the menstrual cycle in both women and macaques, mucosal barriers of mucus and epithelium are altered by hormonal fluctuation. Mucus is the first barrier encountered by HIV-1 in the FRT and is known to change in both levels and consistency during the menstrual cycle and in the presence of hormonal contraceptives [12]. The thickness of the squamous epithelial barriers of the vaginal vault fluctuates greatly in response to hormones in the macaque model [13,14]. For example, an increase in progestin levels during the luteal phase of the menstrual cycle, and after high-dose injection of the contraceptive depot-medroxyprogesterone acetate (DMPA), leads to a thinning of the squamous epithelial barrier [15,16]. Whether similar changes take place in women is less clear and currently debated [17–21].

In the macaque model, DMPA treatment induces a progestin dominant state that is known to increase the efficiency of SIV acquisition after vaginal exposure [16,22]. Recent studies in pigtail macaques have also illustrated increased virus acquisition risk during the late-luteal progesterone-dominant and menses phases of the menstrual cycle [23,24]. Until recently, studies in women were less definitive and there was much debate on this issue [5,7,8,25–27]. However, there is now recent and compelling meta-analytical evidence from two separate studies, suggesting that DMPA increases HIV-1 acquisition [28,29]. It is important for the field to gain mechanistic insights into why there may be an increase in viral acquisition during progestin dominant states.

Here, we utilize two macaque vaginal challenge models to characterize changes occurring in the FRT mucosal barriers over the menstrual cycle and after DMPA treatment. Our first model utilizes reproductive tissues from terminal necropsies of rhesus macaques, during the rhesus macaque anovulatory season, that were either DMPA-treated or untreated. Our second model assesses reproductive tissues from the pigtail macaques either through necropsied SHIV (SF162p3)-infected animals during various phases of the menstrual cycle or SHIV-infected and non-infected vaginal biopsies taken from macaques after DMPA administration. Additionally, nine SHIV-negative pigtail macaque animals were utilized for mucus transport assays. We believe that the comparison of these two widely utilized models is a strength of this study as it identifies similarities in hormonal impact and allows the results obtained here to facilitate the interpretation of the vast majority of macaque research studies to understand vaginal acquisition of SIV/HIV-1.

The centerpiece of our study is the ability to follow viral particles interacting with mucosal barriers by utilizing capabilities to detect fluorescent HIV-1 particles in tissue, thereby allowing the direct observation of how changes in the mucosal barriers influence viral entry paths. [30]. By comparing the barrier function and resident target cell populations of the FRT, we can detect conditions during the progestin-dominant state that could increase the possibility of virus reaching target cells within the lumen. Additionally, by obtaining pigtail macaque cervical mucus and conducting mucus transport assays, we are able to assess how DMPA treatment affects mucus permissiveness for HIV-1 mobility. These mechanistic insights from both macaque models can increase our understanding of how hormonal changes might increase HIV-1 acquisition risk in women.

## Results

### Thickness of stratum corneum in macaques

It is known that the thickness of the squamous epithelial barriers of the vaginal vault of macaque models can vary greatly in response to hormonal signals [13,31]. Preliminary studies have revealed that changes in epithelial thickness were primarily a consequence of alterations in the thickness of the stratum corneum (SC) equivalent of this non-keratinized squamous epithelium. Therefore, we determined the thickness of this layer by defining the boundary of the spinosum-granulosum after immunofluorescent staining with an anti-adherens antibody to E-cadherin. Through the distribution of these cellular junctions we could distinguish the non-viable SC from the nucleated strata and measure the thickness of this SC to the lumen using previously reported algorithms [21,32].

**Fig 1:**
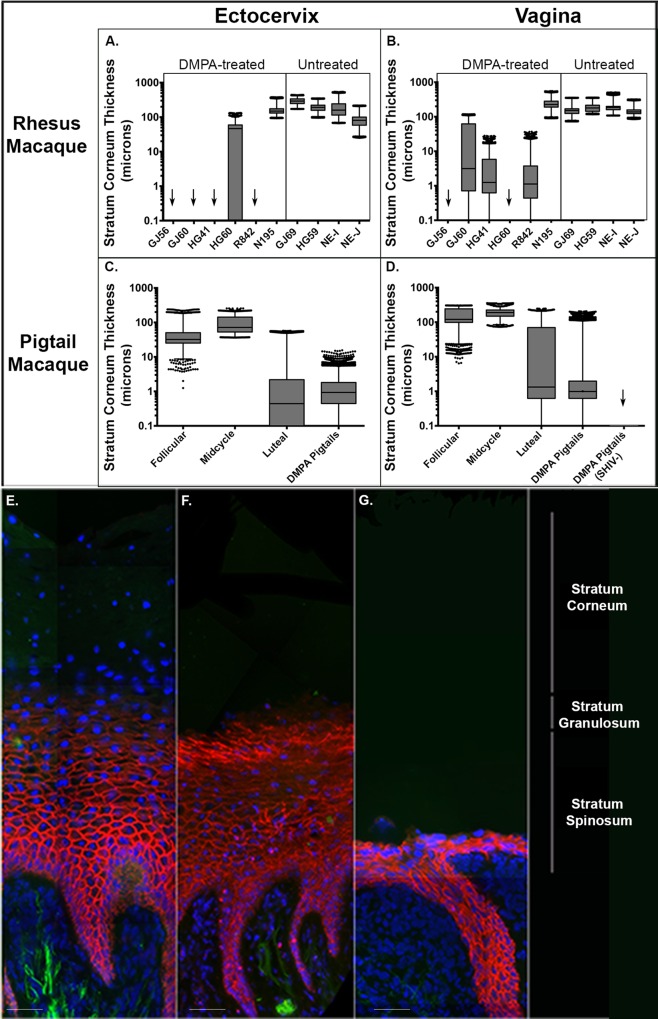
Average Stratum Corneum (SC) Thickness of the Ectocervix and Vagina of Rhesus and Pigtail Macaques. Menstrual cycle phases were designated as follicular (day 1 of menstruation until day 14), midcycle (days 14–16), or luteal (days 17 to day prior to menstruation). DMPA refers to those animals that were pre-treated with intramuscular injections of 30 mg Depo-provera 4–5 weeks (28–33 days) prior to sacrifice. Error bars represent SEM. (a). SC thickness of the ectocervix of progesterone-treated and untreated rhesus macaques. Arrows represent those animals whose SC was absent. (b). SC thickness of the vagina of progesterone-treated and untreated rhesus macaques. Arrows represent those animals whose SC was absent. (c). SC thickness of the ectocervix of infected pigtail macaques by phase of the menstrual cycle and post-DMPA treatment. (d). SC thickness of the vagina of infected pigtail macaques by phase of the menstrual cycle and post-DMPA treatment (30mg). (e). Fluorescent deconvolution image (40x) of the SC thickness in a representative infected pigtail macaque at mid-cycle menstrual cycle phase. Adherens junctions (red), DAPI (blue), tissue background (green). Size bar is 40μm. (f). Fluorescent deconvolution image (40x) of the SC thickness in a representative infected pigtail macaque at luteal menstrual cycle phase pigtail macaque. Adherens junctions (red), DAPI (blue), tissue background (green). Size bar is 40μm. (g). Fluorescent deconvolution image (40x) of the SC thickness in a representative infected pigtail macaque after DMPA treatment. Adherens junctions (red), DAPI (blue), tissue background (green). Size bar is 40μm. Tissue layers are described to the right of image.

When comparing high-dose DMPA-treated (30 mg) with untreated rhesus macaques, the majority of treated animals exhibited an absence of an ectocervical SC layer and a large decrease in vaginal SC thickness (Fig 1A and 1B). Exceptions to this were two DMPA-treated rhesus macaques, HG60 and N195. Both macaques retained thicker ectocervical SC, while N195 also possessed a thick vaginal SC (Fig 1A and 1B).

Likewise, in pigtail macaques during the progestin dominant luteal phase, or after DMPA treatment, obvious reductions in the ectocervical and vaginal SC thickness were observed (Fig 1C–1G. In the luteal phase of pigtail macaques, regions without any apparent SC were visible and after DMPA treatment the spinosum-granulosum could be as thin as 2–3 cell layers (Fig 1F).

### Target cell distribution in macaques

**Fig 2:**
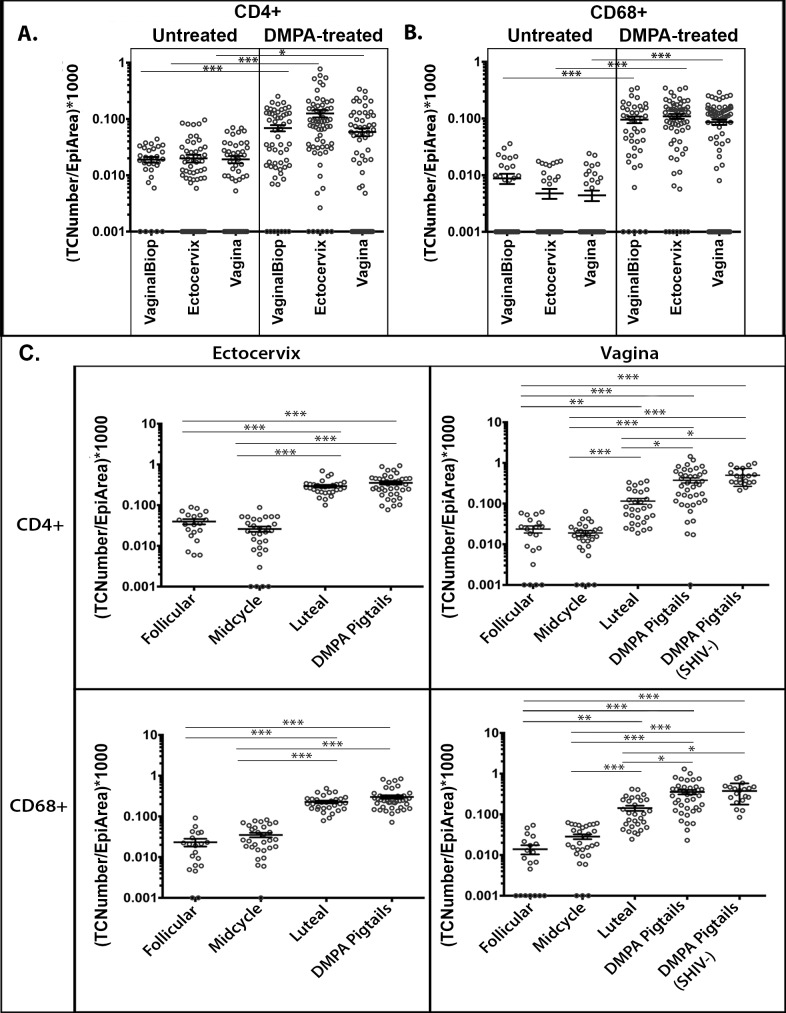
Comparison of Target Cell Density in Various Tissue Types of Rhesus and Pigtail Macaques. Menstrual cycle phases were designated as follicular (day 1 of menstruation until day 14), midcycle (days 14–16), or luteal (days 17 to day prior to menstruation). DMPA refers to those animals that were pre-treated with intramuscular injections of 30 mg Depo-provera 4–5 weeks (28–33 days) prior to sacrifice. TCNumber/EpiArea refers to the number of target cells divided by the area of the epithelium analyzed. Each data point represents the mean cell density from a 40x panel image. Each animal had 10 panel images, 1 panel per random section, taken per tissue type from multiple blocks when available. Error bars represent SEM. (a). Analysis of CD4+ T-cell density in untreated (n = 4) and DMPA-treated rhesus macaques (n = 6), comparing vaginal tissue biopsies and terminal tissue collections (Ectocervix and Vagina). (b). Analysis of CD68+ macrophage density in untreated (n = 4) and DMPA-treated rhesus macaques (n = 6), comparing vaginal tissue biopsies and terminal tissue collections (Ectocervix and Vagina). (c). Analysis of CD4+ T-cell and CD68+ macrophage density in the squamous epithelium of infected pigtail macaques by phase of the menstrual cycle, with comparison to 28–33 day post-DMPA treatment in vaginal biopsies of SHIV infected and non-infected animals: (Top left) CD4+ T-cell density in the ectocervix, (top right) CD4+ T-cell density in the vagina, (bottom left) CD68+ macrophage density in the ectocervix, (bottom right) CD68+ macrophage density in the vagina.

During initial analysis, we noticed an increase in the density of CD4+ T-cells within the squamous epithelium of DMPA treated rhesus macaques, results that were unexpected. Following, we quantified the density of CD68+ and CD4+ target cells located within the stratum malpighii of the ectocervical and vaginal squamous epithelium (Fig 2, S1 Fig, S2 Fig). In high-dose DMPA-treated rhesus macaques, higher densities of CD4+ and CD68+ target cells were found within the epidermis of the ectocervix (both *P≤*0.001) and vagina (*P* = 0.034 and *P≤*0.001, respectively), compared to untreated macaques (Fig 2A and 2B). This difference of CD4+ and CD68+ target cell infiltration was also evident within vaginal biopsy samples, indicating that intra-epithelial cell migration was not a product of PA-GFP HIV-1 administration (both *P≤*0.001). In high-dose DMPA-treated pigtails, ectocervical and vaginal samples contained more intra-epithelial CD4+ and CD68+ target cells than were found during the follicular and mid-cycle phases, regardless of SHIV infection status (all *P<*0.001). Similarly, macaques sacrificed in the late-luteal phase contained more ectocervical and vaginal intra-epithelial CD4+ and CD68+ target cells than found during the follicular and mid-cycle phases (both *P≤*0.001)(Fig 2C, S1 Fig, S2 Fig). This analysis revealed that all macaques in a progestin-dominant state had a statistically significant higher density of CD4+ and CD68+ target cells in the ectocervical squamous epithelium. A significant increase in the density of CD4+ and CD68+ target cells in the vaginal tissue of the pigtail macaques was also observed in the late-luteal phase and after high-dose DMPA treatment, in both SHIV and non-infected animals (Fig 2C, S1 Fig, S2 Fig). In contrast, examination of the CD4+ and CD68+ target cell density in the superficial aspects of the endocervix showed no statistically significant difference over the menstrual cycle in these same animals (S3 Fig).

### Particle diffusion in the FRT mucus of DMPA treated macaques

**Fig 3:**
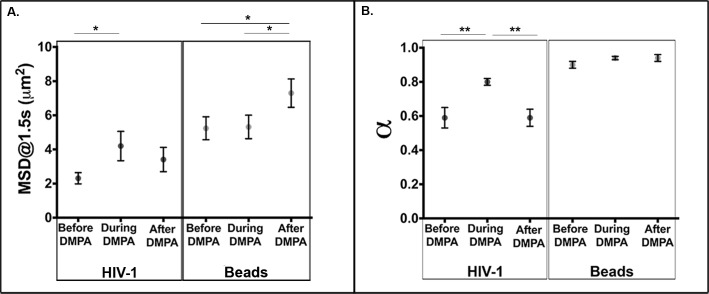
HIV-1 and 200nm bead diffusion in pigtail macaque mucus before, during and after administration of DMPA. Estimated means and standard errors were calculated for (a). Mean-squared displacement (MSD) values for all particle types in each mucus sample collected before (n = 29), during (n = 16) and after (n = 34) DMPA administration. (b). Alpha (α) values for all particle types in each mucus sample collected before (n = 29), during (n = 16) and after (n = 34) DMPA administration. For each treatment condition, generalized linear mixed models were run to compare estimated means for particle MSD and α.

Next we determined differences in virus particle and nanobead diffusion in FRT mucus collected from pigtail macaques before, during, and after injection with DMPA (Fig 3). Tracking the time-dependent trajectory of individual particles allowed the determination of the mean squared displacement (MSD) for HIV and pegylated nanobead. MSD provides information relating to the diffusion constant of particles under different conditions. For beads, the estimated MSD was greatest after DMPA treatment (7.30 μm^2^) when compared to before (5.24 μm^2^, *P* = 0.030) and during treatment (5.32 μm^2^, *P =* 0.033) (Fig 3A). Additionally, the estimated MSD of HIV-1 during DMPA treatment was significantly higher (4.20 μm^2^, *P* = 0.04) when compared to HIV before treatment (2.31 μm^2^). These results reveal that the virus can move through mucus of FRT more easily during the progestin-dominant state induced by high-dose DMPA treatment.

To better characterize the nature of particle mobility, we fit the MSD as a function of lag-time (Δt) to a power law and obtained alpha (α), the diffusion exponent. Again, estimated means and standard errors were calculated for α values for both particle types in each sample collected before, during and after DMPA treatment. An α value equal to 1 indicates free diffusion while a lower α signifies increased obstruction of particle motion. Bead mobility remained nearly unhindered across all conditions such that the calculated estimated means were 0.90, 0.94 and 0.94 for the before, during and after DMPA treatment conditions, respectively. In contrast, HIV-1 mobility was significantly freer during DMPA treatment (0.80) when compared to before (0.59, *P* = 0.008) and after (0.59, *P* = 0.003) (Fig 3B). These results demonstrate that DMPA treatment increases mucus permissiveness for HIV-1 mobility over timescales of 1.5s.

### PA-GFP HIV-1 tissue association in macaques

**Fig 4:**
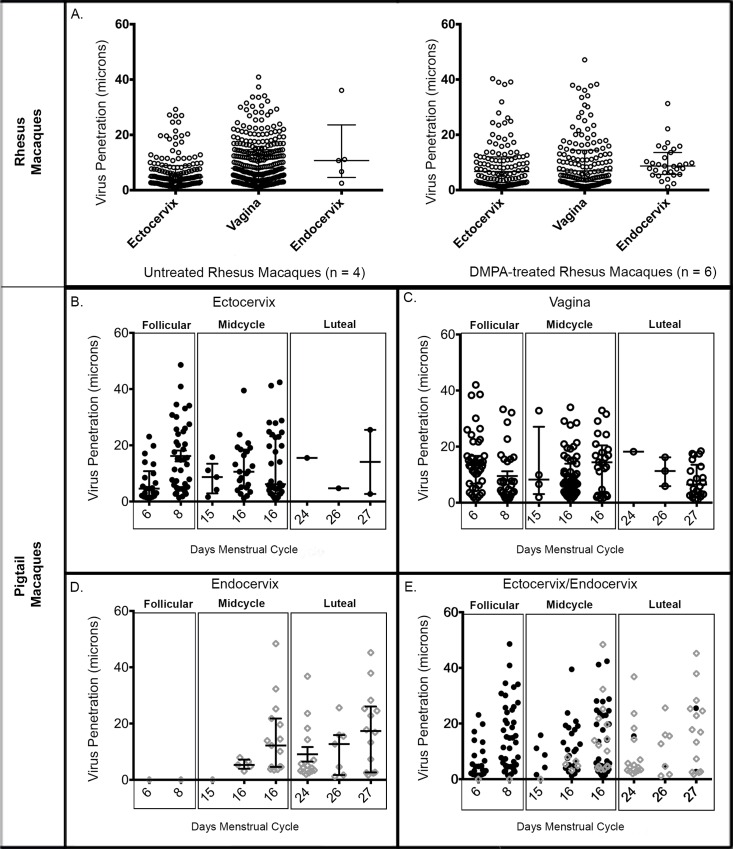
*In Vivo* PA-HIV-1 Penetration of Rhesus and Pigtail Macaque Genital Tracts. Each data point displayed represents an individual penetrating virion. Each animal had ~20 100x images for each available block of each tissue type. Error bars represent SEM. (a). (Left) Virus penetration in the female reproductive tract of untreated rhesus macaques. (Right) Virus penetration in the female reproductive tract of progesterone-treated rhesus macaques. (b). Virus penetration of the ectocervix of infected pigtail macaques (n = 8) by day of the menstrual cycle. (c). Virus penetration of the vagina of infected pigtail macaques by day of the menstrual cycle. (d). Virus penetration of the endocervix of infected pigtail macaques by day of the menstrual cycle. (e). Virus penetration of the ectocervix (b) and endocervix (d) combined. n = number of animals.

In order to assess *in vivo* viral tissue association, all macaques except DMPA-treated pigtail macaques, were intravaginally inoculated with high titer PA-GFP HIV-1 and euthanized 4 hours post-inoculation. In these tissues, the majority of samples from both DMPA treated and untreated rhesus macaques and cycling pigtail macaques exhibited PA-GFP virions associated with each tissue type, between epithelial cells, and penetrating the squamous and columnar epithelial barriers up to depths of 50um, potentially within reach of target cell populations (Table 1, Fig 4, S4 Fig, S5 Fig).

**Table 1:** Analysis of PA-GFP HIV-1 in rhesus macaques with and without DMPA treatment and pigtail macaques at different menstrual cycle phases.

Animal	Animal Model	Tissue Type	z-Scans (z)	Virions (v)	v/z	Penetrators (p)	p/z	Avg. Depth (μm)	Max. Depth (μm)
**Rhesus Macaques**	**PA-GFP HIV-1 Penetration: in vivo untreated rhesus macaques n = 4**	**Ectocervix**	505	356	0.70	157	0.31	6.9	29.2
		**Endocervix**	540	9	0.02	5	0.01	13.4	36.1
		**Vagina**	1067	1109	1.04	294	0.27	10.3	40.9
	**PA-GFP HIV-1 Penetration: *in vivo* DMPA treated rhesus macaques n = 6**	**Ectocervix**	1006	343	0.34	120	0.12	9.2	40.3
		**Endocervix**	759	51	0.07	31	0.04	9.9	31.3
		**Vagina**	1058	294	0.28	140	0.13	10.5	47.1
**Pigtail Macaques**	**PA-GFP HIV-1 Penetration: *in vivo* pigtail macaque, follicular n = 2**	**Ectocervix**	329	94	0.29	62	0.19	13	29.2
		**Endocervix**	315	15	0.05	0	0	0	0
		**Vagina**	446	141	0.32	101	0.23	12.9	41.3
	**PA-GFP HIV-1 Penetration: *in vivo* pigtail macaque, midcycle n = 3**	**Ectocervix**	522	85	0.16	64	0.12	12.4	42.4
		**Endocervix**	517	37	0.07	20	0.04	11	32.9
		**Vagina**	705	144	0.2	86	0.12	12.5	48.4
	**PA-GFP HIV-1 Penetration: *in vivo* pigtail macaque, luteal n = 3**	**Ectocervix**	481	17	0.04	4	0.01	12.1	25.5
		**Endocervix**	495	62	0.13	35	0.07	12.8	45.2
		**Vagina**	610	54	0.09	26	0.04	8.4	18.4

Differences in virus tissue association were observed between DMPA treated and untreated rhesus macaques (Table 1). In the ectocervix of untreated rhesus macaques, analysis of 505 z-stack images revealed 356 PA-GFP HIV-1 particles associated with the tissue. In DMPA treated macaques, analysis of 1006 z-stack images illustrated only 343 PA-GFP HIV-1 particles interacting with the tissue. Although there were no differences in PA-GFP HIV-1 penetration between untreated and DMPA treated macaques (*P =* 0.997), there was slight increase in the number of virions associated with the surface of the ectocervical stratum corneum in untreated animals (*P* = 0.296), although not statistically significant. Similarly, in the vagina, there was a slight increase in virion association with the squamous epithelium (*P* = 0.127). After analyzing 1067 z-stacks, 1109 virions were found to interact with the squamous epithelium in untreated rhesus macaques, compared to 294 virions in 1058 z-stacks in DMPA treated macaques. However, unlike what was noted in with the ectocervix, there was a significant increase in the number of penetrating virions per z-scan in untreated macaques when compared to those animals that were DMPA treated (*P*<0.001), results suggesting that the tissues of the ectocervix and vagina may not be as similar as previously posited. Inversely, DMPA-treated macaques depicted a significant increase in the number of virions entering the endocervical canal and interacting with the simple columnar tissue, compared to untreated macaques (*P* = 0.003), although there was no difference in the number of penetrating virions between the two groups (*P =* 0.212).

To extend this work to address natural hormonal changes over the menstrual cycle, we conducted complementary studies using PA-GFP HIV-1 in the *in vivo* pigtail macaque model. However, one caveat to this particular study was that we were only able to gain access to a small number of SHIV (SF162p3)-infected animals. This small sample size of animals thereby impeded statistical analysis and has been described in more detail in the materials and methods. Regardless, similar to rhesus macaques that received exogenous progestin, pigtail macaques in the high progestin, late-luteal phase of the menstrual cycle exhibited more virions associated with the epithelium of the endocervical canal (Fig 4B–4E, Table 1). Of 495 images, 62 virions were associated with the simple columnar, of which 57% (*n =* 35) were penetrating (Fig 4D). However, of 315 images, no virions were seen penetrating the simple columnar of the progesterone-low follicular stage. Interestingly, during the mid-cycle period, when progesterone begins to rise, 37 virions amongst 517 images were located within the canal. Of these, 54% (*n =* 20) penetrated the simple columnar (Fig 4D). Most of these penetrating virions were seen in a single animal. Moreover, much like the DMPA-treated rhesus macaques, there were also fewer virions associated with the squamous epithelia of the ectocervix and vagina during the late-luteal phase. In contrast, more virions interacted with the squamous epithelium of the ectocervix and vagina of those macaques in the progesterone-low follicular and mid-cycle stages. In the follicular phase, 66% (*n =* 62) of identified virions penetrated the ectocervical epithelium (Fig 4B) and 72% (*n =* 101) penetrated the vaginal epithelium (Fig 4C). During mid-cycle, 75% (*n =* 64) of identified virions penetrated the ectocervical epithelium (Fig 4B) and 60% (*n* = 86) penetrated the vaginal epithelium (Fig 4C).

## Discussion

The studies presented here were designed to gain mechanistic insights into increased HIV-1 acquisition associated with vaginal challenge in macaque models. The FRT is a hormonally sensitive mucosal environment with dynamic changes in mucosal barrier function. Macaque models have revealed that progestin-dominant hormonal states, both endogenous and exogenous, can increase the efficiency of SIV/SHIV acquisition after vaginal challenge [16]. HIV-1 transmission requires that the virus in the inoculum reach a susceptible target cell in the mucosal tissue. We observed two progestin-dominant state changes in the mucosal barriers of macaques that would increase the likelihood of HIV-1 interacting with tissue resident target cells compared to during low-progestin states: increased viral entry into the endocervical canal and increased proximity of virus to target cells infiltrating the squamous epithelium.

Utilizing the live pigtail macaque challenge model, we found that a paucity of virus associated with the columnar epithelium during the follicular phase of the menstrual cycle. The same held true for rhesus macaques that were out-of-season and non-cycling, a state also defined by minimal progestin levels. In contrast, after vaginal challenge during progestin-dominant states we observed an increase in virions able to reach the endocervical columnar epithelial barrier. This was noted, after high-dose DMPA treatment, for both rhesus and pigtail macaques as they entered the menstrual cycle luteal phase.

The ability of the virus to better reach the endocervical columnar epithelium during progestin dominant states might be due to anatomical changes, alterations in mucus function, or a combination of the two [33,34]. Progestin-based hormonal contraceptives, such as DMPA, influence mucus by drastically reducing volume production and increasing viscosity to prevent sperm access to the cervical canal [35]. The direct analysis of HIV-1 mobility in pigtail macaque FRT mucus revealed that the ability of HIV-1 to diffuse through FRT mucus was increased relative to the diffusion of the pegylated nanobeads during DMPA treatment (Fig 3). The pegylated nanobeads are believed to be resistant to charge-based interactions with mucins (muco-adhesion), and instead measure mucus pore size [36]. Consistent with hormonal changes influencing the muco-adhesion of the viral particles, we see minimal influence of the hormonal treatment on the mobility of the beads, which are non-muco-adhesive and of a similar size to the viral particles. These observations do not point to mucus inhibition of HIV-1 mobility by simple changes in pore size but suggest a virus-specific impediment for HIV-1 that is significantly abrogated in the presence of exogenous progestin. We have previously detected this type of activity in human cervicovaginal mucus [37]. The reported influence of pH on virus mobility further supports the potential role of muco-adhesion in this system [38–40].

**Fig 5:**
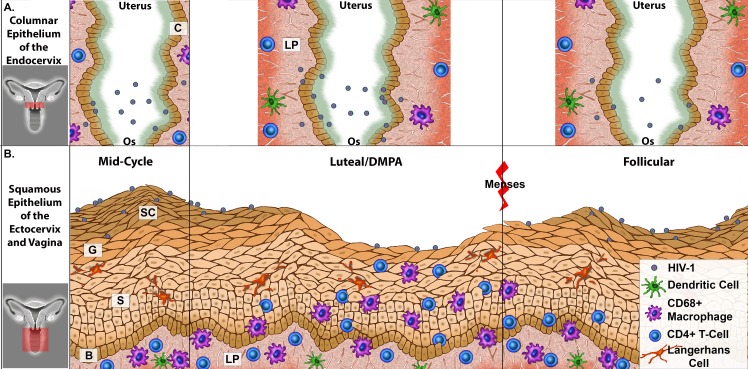
Mechanistic Model of How HIV-1 Interacts with the Female Reproductive Tract during Various Phases of the Menstrual Cycle and/or with the use of DMPA. (a). The simple columnar epithelium of the endocervix: (Left) as mid-cycle progresses into luteal phase, a small number of virions are able to enter the cervical canal to associate with the simple columnar epithelium of the endocervix. (Middle) During the luteal phase and/or with the use of progesterone contraceptives (e.g., DMPA), virions are more readily able to enter the cervical canal to interact with the simple columnar epithelium of the endocervix. (Right) During the follicular phase, virions rarely enter the cervical canal to interact with the endocervical simple columnar. Tissue layers are labeled as follows: C = simple columnar, LP = lamina propria. (b). The stratified squamous epithelium of the ectocervix and vagina: (Left) At the midcycle phase, the squamous epithelium is thickest and virions may penetrate the non-viable stratum corneum. However, target cells are primarily located below the epidermis, in the lamina propria; therefore, the likelihood of virus interacting with a target cell is minimal. (Middle) Although few virions are visualized penetrating the stratified squamous epithelium due to the lack of stratum corneum during the luteal phase (or with exogenous progesterone treatments; e.g., DMPA), there is a greater propensity for virus to interact with an infiltrating intraepithelial target cell near the luminal surface. (Right) Like the mid-cycle phase, the squamous epithelium during the follicular phase is thick, and virions are associated with the non-viable stratum corneum. However, target cells are primarily located below the epidermis, in the lamina propria; therefore, the likelihood of virus interacting with a target cell is minimal. Tissue layers are labeled as follows: SC = stratum corneum, G = granulosum, S = spinosum, and B = basal layer, LP = lamina propria.

The results presented in Fig 3 support the hormonal regulation of this muco-adhesion. During hormone treatment there is a significant increase in the MSD and α of HIV-1 in the absence of any change in the bead mobility. Further support for hormonal influence is seen during the washout period where the MSD of the beads increases significantly while the MSD and α of HIV-1 is decreased. It is known that mucins are highly glycosylated and alterations in these glycosylations are hormonally regulated, which may be responsible for anchoring pathogens to mucus [41–43]. For example, DMPA has been shown to reduce expression of negatively charged sialic acid carbohydrates on these proteins; therefore, there may be natural repulsion that occurs between HIV-1 and these carbohydrates in mucus, providing favorable conditions for HIV-1 transport. [43]. Importantly, the increased HIV-1 diffusion through mucus observed after DMPA treatment was associated with increased virus entry into the endocervical canal during the progestin-dominant state. This increased ability of virus to enter the endocervical canal and reach the columnar epithelial barrier is likely part of the mechanism by which HIV-1 acquisition may be increased during high-progestin environments (Fig 5).

Changes in the squamous epithelium of the vaginal vault during progestin-dominant states also increased the likelihood that virus might encounter and potentially infect tissue resident target cells. The most obvious change in the squamous epithelium was the decrease in the epithelial barrier thickness of the vaginal vault during the natural or induced progestin-dominant state (Fig 1) [13–15,44]. The ability of HIV-1 to penetrate the squamous epithelium did not appear to be associated with a decrease in epithelial thickness, but rather was more readily observed in thicker SC [30]. This phenomenon can be especially noted within the ectocervix of HG60 and the ectocervix and vagina of N195; two DMPA-treated rhesus macaques that retained a thick SC and exhibited a large number of penetrating HIV-1 virions (Fig 1, S5 Fig). However, in the case where virus can enter the non-viable pseudo-keratinized layer found in untreated animals, there were no target cells within this non-viable layer. Therefore, when progestin influence was minimal, the thick SC offered a partial barrier to HIV-1 transmission by increasing the distance that virions must traverse to reach viable target cells.

The decreased thickness of the squamous epithelial barrier during the progestin-dominant state was associated with an increase in the number of CD4+ T-cells and CD68+ macrophages within the squamous epithelium. Previous studies have suggested no difference in target cell distribution in progesterone-treated macaques or during different menstrual cycle phases [45,46]. However, unlike the studies here, they did not strictly focus on the epidermis or only looked at CD3+ cells [47]. Here we focused on target cell density within the squamous epithelium and observed a significant increase in intra-epithelial CD4+ T-cells and CD68+ macrophages during the natural and DMPA-induced progestin-dominant state. Additionally, in DMPA-treated pigtail macaques, there were significant increases in intra-epithelial target cells regardless of macaque SHIV status, results that suggest that target cell infiltration is a product of a high-progesterone environment and not lentiviral infection (Fig 2C). Interestingly, we observed two rhesus macaques (HG60 and N195) that exhibited characteristics similar to both untreated and DMPA-treated rhesus macaques. Although both animals were treated with DMPA, the SC of the ectocervical (HG60 and N195) and vaginal squamous epithelium (N195 only) remained present and thick, akin to untreated macaques. Furthermore, in those tissues where the SC remained thick, both rhesus macaques displayed a large number of virions “stuck” within the SC of the squamous epithelium and a decreased density of intraepithelial target cells results that also mirrored the untreated group and affected the overall statistics of the combined DMPA-treated animals (S1 and S5 Figs). However, these macaques also illustrated viral association with the endocervical simple columnar epithelium similar to what was observed in DMPA-treated macaques (S5 Fig). These data suggest two conclusions: 1) it is the epithelial physiology, which can be manifested in a variety of ways (thinning, cellular junction alteration, etc.), and not progesterone directly, that affects intraepithelial target cell infiltration, and 2) progesterone can still affect mucus permeability properties. This would indicate there are different progestin-influenced pathways in the FRT for changes in mucus function versus alterations in epithelial physiology.

It is notable that infiltration of target cells into the stratum malpighii would bring them closer to the lumen. Target cells close to the lumen were prominent in the squamous epithelium during the progestin-dominant state. Interestingly, when comparing DMPA-treated to untreated rhesus macaques, virus penetration was less apparent within the squamous tissues during the progestin-dominant state. Whether this is due to virus clearing by accessible target cells or being endocytosed by live keratinocytes of the stratum malpighii is not known. However, this situation, where the potential target cells are coming to where the virus is located, is potentially a mechanism for the increased acquisition observed in the progestin-dominant state. In the macaque model, CD4+ T-cells and CD68+ macrophage distribution, and virus penetration were dependent on epithelial thickness, suggesting HIV-1 interactions with the FRT may differ in the luteal and follicular stages of the menstrual cycle or with the use of progesterone-only contraceptives (Fig 5). Overall, the results presented here reveal that the high-dose DMPA models and the natural luteal phase in the pigtail macaque model show general similarity.

How these observations in both macaque models relate to potential hormonal influences of increased acquisition in women remain to be elucidated. For example, it is clear that the standard high-dose DMPA treatment used here is much higher than the levels utilized in women. It has recently been reported that DMPA doses in the pigtail macaque model similar to the dose for women results in much smaller effects on epithelial thickness [47]. Previous studies of squamous epithelial thickness suggest that there is little to no variability in FRT epithelial thickness in women with exogenous or endogenous progestin exposure [48,49]. However, other studies suggest that differences in epithelial thickness can be detected [21]. Similar discrepant results have been obtained relating to target cell density in the squamous epithelium. Some clinical studies in women suggest that target cell density does not change [18,49–51], while other studies have suggested that the vaginal CD4+ T-cell and CD68+ macrophage populations increased significantly amongst women receiving DMPA injections [48,52]. Likely, differences in analytical methods and sampling times account for these differences.

If we extrapolate the macaque studies to HIV-1 acquisition in women, it suggests that HIV-1 acquisition can be influenced by menstrual cycle and hormonal contraceptives. To this end it should be possible to study the epithelial thickness, target cell localization, and mucus barrier function in women over the menstrual cycle and during the use of hormonal contraceptives. Considering the building consensus that DMPA can increase HIV-1 acquisition in women, it seems likely that some of the mechanisms identified here are associated with higher acquisition risk, including increased virus association with the endocervical columnar barrier and the infiltration of target cells into the squamous epithelium during the progestin dominant state. These studies provide mechanistic insight into the changes of physiological parameters of the FRT under various hormonal conditions associated with increased risk of acquisition and should be considered in the development of novel HIV-1 prevention technologies.

## Materials and Methods

### Study design

We received access to 8 pigtail macaques (Centers for Disease Control and Prevention) infected with the non-pathogenic SHIV strain SF162p3 and examined animals in an exploratory study in three distinct stages of the menstrual cycle. The study was descriptive and therefore not powered. Animal handlers and laboratory staff performing assays were aware of each animal’s phase in the menstrual cycle, but the samples were shipped to the Hope Laboratory for evaluation in blinded manner. The tissues for all SHIV (SF162p3)-infected cycling animals were examined for epithelial thickness, virus penetration, and target cell density. Likewise, four SHIV (SF162p3)-infected pigtail macaques were pre-treated with intramuscular injections of 30 mg Depo-provera/DMPA 4–5 weeks (28–33 days) prior to sacrifice. These samples, along with vaginal biopsies from 2 DMPA-treated non-infected pigtail macaques were also assessed for epithelial thickness and target cell density. Complementary studies were done in 10 rhesus macaques (Tulane National Primate Research Center) comparing DMPA treated and control animals. Additionally, mucus studies in 9, SHIV-negative, pigtail macaques were also conducted; focusing on virus particle and nanobead mobility in DMPA treated and untreated animals. All animal experiments were conducted in accordance with each facility’s respective Institutional Animal Care & Use Committee (IACUC) guidelines. No animals were excluded from analyses.

### Virus development and characterization

All R5-HIV-1_Ba-L_ PA-GFP-Vpr viral stocks were created and characterized as previously described [30].

### Ethics statement

All macaques were housed at the Tulane National Primate Research Center (TNPRC) (*Macaca mulatta*) or the Centers for Disease Control and Prevention (CDC) (*Macaca nemestrina)*, both in accordance with the Association for Assessment and Accreditation of Laboratory Animal Care International standards. All rhesus macaque studies were reviewed and approved by the Tulane University Institutional Animal Care and Use Committee under protocol number P0153. Additionally, all pigtail macaque studies were reviewed and approved by the Institutional Animal Care and Use Committee of the Centers for Disease Control and Prevention under protocols 2445, 2009, and 2423. All animal housing and studies were carried out in strict accordance with the recommendations in the Guide for the Care and Use of Laboratory Animals of the National Institutes of Health (NIH, AAALAC #000594) (TNPRC) or the National Research Council (U.S.) Committee for the Update of the Guide for the Care and Use of Laboratory Animals Institute for Laboratory Animal Research (U.S.), National Academies Press (U.S.), Guide for the care and use of laboratory animals. 8th ed. Washington, D.C.: National Academies Press, 2011 (CDC), both with the recommendations of the Weatherall report; “The use of non-human primates in research”. Rhesus macaques were provided ad libitum with Monkey chow (Lab Fiber Plus Primate diet-DT, PMI Nutrition International, St. Louis, MO) and supplemented with fruits, vitamins and Noyes’ treats (Research Diets, New Brunswick, NJ). Pigtail macaques were provided with an assortment of food selections such as fruits, vegetables, or seeds. All clinical procedures were carried out under the direction of a laboratory animal veterinarian. All procedures were performed under anesthesia using ketamine, often in combination with telazol, and all efforts were made to minimize stress, improve housing conditions, and to provide enrichment opportunities (e.g., objects to manipulate in cage, varied food supplements, foraging and task-oriented feeding methods, interaction with caregivers and research staff). Euthanasia of all macaques were performed in a humane manner (intravenous pentobarbital) as recommended by the American Veterinary Medical Association Guidelines on Euthanasia, 2013, and in accordance with the euthanasia policies of each institution.

### Macaques

Six rhesus macaques were pre-treated with intramuscular injections of 30 mg depo-medroxyprogesterone acetate (Depo-provera/DMPA) 28–33 days prior to virus exposure, leaving 4 macaques untreated. Approximately, 4mL of high titer PA-GFP HIV-1 was intravaginally applied to all anaesthetized animals that were subsequently returned to their cages for 4 hours. Likewise, four SHIV (SF162p3)-infected and two non-infected pigtail macaques were pre-treated with intramuscular injections of 30 mg Depo-provera /DMPA 4–5 weeks (28–33 days) prior to sacrifice or prior to vaginal biopsy acquisition, respectively. SHIV (SF162p3) is a nonpathogenic virus that results in a very low viral load, and does not cause a permanent decrease in CD4+ T-cell populations. For menstrual cycle-related studies, eight SHIV (SF162p3) -infected pigtail macaques were visually inspected for perineal tumescence and menses onset and progesterone measurements taken, as previously described, to determine the approximate day of menstrual cycle prior to HIV-1 exposure [24,53]. 1mL of PA-GFP HIV-1 was intravaginally applied at pre-determined days/phases of the menstrual cycle. Phases were designated as follicular (day 1 of menstruation until day 14), midcycle (days 14–16), or luteal (days 17 to day prior to menstruation). All macaques were euthanized and PA-GFP HIV-1 inoculated reproductive tracts were removed for analysis. Multiple 1cm^3^ samples were harvested from the ectocervix, endocervix, and vagina. Tissue samples were preserved in optimal cutting temperature (OCT) and stored at -80°C.

### Mucus collection

To assess the effects of contraceptives on HIV-1 transport in mucus, nine additional SHIV-negative pigtail macaque animals were utilized [54]. During the entirety of the study, all mucus samples were collected once weekly for all animals. To establish a baseline, mucus was collected for 4–6 weeks prior to intramuscular DMPA administration, thereby comprising the “before” condition. Macaques were divided into 3 groups of n = 3 and received either a 0.5, 1.5 or 2.5mg/kg dose of DMPA once every 4 weeks for 2 months. Mucus was collected weekly, to up to 4 months after the final DMPA injection. Samples collected from first injection up to one month after the final injection made up the “during” condition. The after condition consisted of all sample collections occurring one month after final DMPA injection until the end of the study (~2 months) For analysis, animals receiving different DMPA doses were pooled.

During collection, the cervix was exposed using a vaginal speculum and cervical mucus was collected using a sterile Aspirette (Cooper Surgical) by insertion into the cervical os, approximately 1.5 cm or less with applied negative pressure. Cervical secretions were then expelled into a sterile 1.5ml Eppendorf O-ring tube (Fischer Scientific) and placed on ice until imaging the next day after shipment to Chicago, IL.

### HIV-1 and nano-bead mucus transport assay

Particle diffusion mucus transport assays were performed in accordance with established protocols as previously described [37]. Concentrated R9 BaL Gag-cherry (HIV-1) and fluorescent polystyrene 200nm nanobeads were mixed in equal concentration [37]. Aliquots of aspirated macaque vaginal mucus (5μL) were pipetted onto glass slides with affixed double-sided adhesive single-well spacers. To the mucus aliquot center, 0.5μL of HIV-1/beads mixture was added and gently pipetted until uniformity was achieved. Coverslips were placed over the mucus and secured with nail polish. Movies of both particle types were imaged inside of a temperature-controlled chamber maintained at 37°C with an EMCCD camera, attached to a deconvolution microscope. Imaging occurred 30μm away from the coverslip and no less than 100 particles of each type were imaged every 150ms for one minute. Individual particle positions for each particle type were analyzed using two-dimensional custom-based algorithmic particle tracking software. The collective population mobility was measured by calculating the time-averaged ensemble mean squared displacement (MSD) over timescales of 1.5 seconds (Δt of 1.5s) using custom-based particle tracking software to determine the individual particle positions for HIV and the muco-inert nanobeads [37,55].

### Immunofluorescence

Sectioned tissues were fixed in 3.7% formaldehyde in PIPES buffer and blocked with normal donkey serum prior to staining. For adherens junction identification in macaque tissues, HECD1 (a gift from the laboratory of Dr. Kathy Green at Northwestern University) was utilized. To identify target cells, rhesus macaque tissue was stained with MCD1 (Santa Cruz) CD4 (Cell Marque) and CD68 for macrophages (DakoCytomation). Additional antibodies revealed cytokeratin-7 (DakoCytomation) staining in simple columnar tissue. To confirm that all PA-GFP fluorescence was associated with viral proteins, p24 (AG3.0 National Institutes of Health AIDS Research and Reference Reagent Program; Jonathon Allan), and p17 (Capricorn) antibodies were used [56]. Secondary antibodies, Rhodamine RedX (Jackson ImmunoResearch) and Cy5 (Jackson ImmunoResearch), were also utilized. Antibody specificity was determined by negative results with respective isotype control antibodies. Hoechst DAPI (Invitrogen) was used for DNA staining and wheat germ agglutinin (Invitrogen) highlighted cellular glycoproteins. After staining, mounting medium (DakoCytomation) and coverslips were applied and sealed with nail polish.

### Imaging and image analysis

Images were obtained by deconvolution microscopy on a DeltaVision RT system collected on a digital camera (CoolSNAP HQ; Photometrics) using a 40x or 100x oil objective. To assess epithelial thickness in rhesus and pigtail macaques, we took ten measurements of each tissue type per macaque. By utilizing an anti-adherens antibody, we distinguished the stratum corneum of each sample from the viable layers of the squamous epithelium. Using IDL and specifically created algorithms we measured the stratum corneum thickness, as previously described [21].

For virus penetration analyses, 100x Z-scan stacks were collected over 15μm for each image field and image analysis was performed with the softWoRx software (Applied Precision). Several criteria were developed to assure the visualization of photoactivated virions, including a minimum of 2-fold increase in intensity post-photoactivation. Post-photoactivation GFP signal intensity was measured using softWoRx line profiling software. Penetrating virions were defined as virions entering more than one micron into the squamous or columnar epithelium and were determined with the measuring tool supplied by the softWoRx software. We use the cut off of 1 micron due to the resolution of our system. With a measurement less than 1 micron, we cannot be sure if the virion is in the tissue or on the surface of the tissue; therefore, 1 micron and beyond ensures that the detected virion is indeed penetrating. For each tissue block, 20 images were obtained with locations chosen randomly from multiple tissue sections. All combined tissue blocks encompassed the entire cervix and vaginal vault of each animal ensuring that any variability in installation would not be problematic and also allowing us to detect viral particles in each tissue type. Regions imaged were 60μm wide and 12μm thick.

To determine target cell density in macaques, we took ten measurements, across multiple tissue blocks, of each tissue type per macaque. Panel images were acquired to include the epithelium and lamina propria. Each image consisted of a stitched panel comprised of three 40x images across the lumen and *n* 40x images to the basal layer, *n* being dependent on the epidermal thickness of each sample. Using the SoftWorX software we calculated areas of robust epithelial adherens junctions and determined target cell densities for each sample. To calculate density, the number of target cells in each sample was divided by the average HECD-1 area. The softWoRx software measuring tool was used to measure the shortest distance of intraepithelial target cells to the lumen.

### Statistical analysis

All virus penetration data were analyzed using data per image or per virion and included three models. The three models were; a negative binomial generalized estimating equation (GEE) modeling of the count per image of virions present on the tissue (all observations per image), a binomial GEE with logit link modeling of the proportion per image of virions that penetrate the tissue (observations with at least 1 virion present per image), and a gamma GEE modeling of the depth of penetration per virion. This approach was applied to two datasets: 1) rhesus macaque *in vivo* examining the covariates DMPA treatment and tissue type (*n* = 8, 4218 images) and 2) pigtail macaque *in vivo* (*n* = 8, 4420 images) examining the covariates menstrual stage and tissue type. Unfortunately, with the pigtail macaque samples, the data were too sparse to calculate the model with the main effects of tissue type (ectocervix, endocervix, and vagina), along with menstrual cycle phase (follicular, midcycle, and luteal) and the interaction of the two variables. Overall, the GEE model was over saturated and the generalized Hessian matrix was not positive definite; therefore, the model would not run.

All comparisons between multiple groups for target cell density analysis were performed. The initial datasets examined intraepithelial cell count for DMPA and untreated rhesus macaques. Later datasets included pigtail macaque data and examined in detail intraepithelial target cell density based on hormonal environment: DMPA treated and phases of the menstrual cycle. All data sets were stratified by tissue type. Target cell count per unit of area was examined in each dataset using negative binomial or zero-inflated negative binomial GEE where appropriate.

Gamma GEE models were performed to assess the relationship between particle dispersion (MSD) and time. Time consists of before, during, and after treatment with repeated measures across days within each time period. The ‘before DMPA’ condition is represented by sample collections occurring four to six weeks before initial DMPA injection. One week after the initial injection and up to one month after the final injection make up the ‘during DMPA’ condition. The final condition, ‘after DMPA’ is comprised of all samples that occurred one month after the final DMPA injection. The models were performed separately for beads and virus particles. The underlying distribution of the outcome was assumed to be a gamma, accounting for repeated measures within monkey.

All analysis were performed using SAS 9.4 and all limits of statistical significance were set at p < 0.05.

## Supporting Information

S1 Fig Comparison of Target Cell Density in Various Tissue Types of Individual Rhesus Macaques. DMPA refers to those animals that were pre-treated with intramuscular injections of 30 mg Depo-provera 4–5 weeks (28–33 days) prior to sacrifice. TCNumber/EpiArea refers to the number of target cells divided by the area of the epithelium analyzed. Each data point represents the mean cell density from a 40x panel image. Each animal had 10 panel images, 1 panel per random section, taken per tissue type from multiple blocks when available. Error bars represent SEM. (a). Analysis of CD4+ T-cell density in untreated (n = 4) and DMPA-treated rhesus macaques (n = 6), comparing terminal tissue collections (Ectocervix and Vagina) and vaginal tissue biopsies. (b). Analysis of CD68+ T-cell density in untreated (n = 4) and DMPA-treated rhesus macaques (n = 6), comparing terminal tissue collections (Ectocervix and Vagina) and vaginal tissue biopsies.
(JPG)

S2 Fig Qualitative Analysis of Target Cell Density in Macaques. Fluorescent deconvolution images (40x) of various macaque samples. CD4+ T-cells (red), DAPI (blue), tissue background (green). Size bars are 40**μ**m. Tissue layers are labeled as follows: SC = stratum corneum, G = granulosum, S = spinosum, and B = basal layer, LP = lamina propria. (a). Vaginal epithelium from a DMPA-treated rhesus macaque (30mg). (b). Vaginal epithelium from a DMPA-treated infected pigtail macaque (30mg). (c). Vaginal epithelium from an untreated rhesus macaque. (d). Vaginal epithelium from the midcycle menstrual cycle phase in an infected pigtail macaque. (e). Vaginal epithelium from the follicular menstrual cycle phase in an infected pigtail macaque. (f). Vaginal epithelium from the luteal menstrual cycle phase in an infected pigtail macaque.
(JPG)

S3 Fig Comparison of Target Cell Density in Endocervical Tissue of Cycling Pigtail Macaques. Menstrual cycle phases were designated as follicular (day 1 of menstruation until day 14), midcycle (days 14–16), or luteal (days 17 to day prior to menstruation). TCNumber/EpiArea refers to the number of target cells divided by the area of the epithelium analyzed. Each data point represents the mean cell density from a 40x panel image. Each animal had 10 panel images, 1 panel per random section, taken per tissue type from multiple blocks when available. Error bars represent SEM. (a). Analysis of CD4+ T-cell density in the simple columnar epithelium of infected pigtail macaques by phase of the menstrual cycle. (b). Analysis of CD68+ macrophage density in the simple columnar epithelium of infected pigtail macaques by phase of the menstrual cycle.
(JPG)

S4 Fig Entry of PA-GFP HIV-1 into the Stratified Squamous Epithelium of the Macaque Vagina. Fluorescent deconvolution images (100x) of macaque vagina. Size bars are 20μm. (a) GFP signal before photoactivation, illustrating tissue autofluorescence (green) and DAPI (blue). (b) GFP signal after photoactivation (pseudo-colored red) and DAPI (blue). (c) An overlay of GFP signal before (A) and after photoactivation (B). Identified virions are shown (red, white arrows), pre-photoactivation (green) and DAPI (blue). (d) Identified virions are shown (red), WGA (green), and DAPI (blue).
(JPG)

S5 Fig Comparison of Virus Penetration in Various Tissue Types of Individual Rhesus Macaques. DMPA refers to those animals that were pre-treated with intramuscular injections of 30 mg Depo-provera 4–5 weeks (28–33 days) prior to sacrifice. Analysis of PA-GFP HIV-1 virus penetration in untreated (n = 4) and DMPA-treated rhesus macaques (n = 6), comparing terminal tissue collections (ectocervix and vagina) and vaginal tissue biopsies. Each data point displayed represents individual penetrating virions. Each animal had ~20 100x images for each available block of each tissue type. Error bars represent SEM.
(JPG)
