# Supplementary figures and images for: Increases in Endogenous or Exogenous Progestins Promote Virus-Target Cell Interactions within the Non-human Primate Female Reproductive Tract
Source: PLoS Pathog. 2016 Sep 22;12(9):e1005885. doi: 10.1371/journal.ppat.1005885 (PMC5033389; doi:10.1371/journal.ppat.1005885)

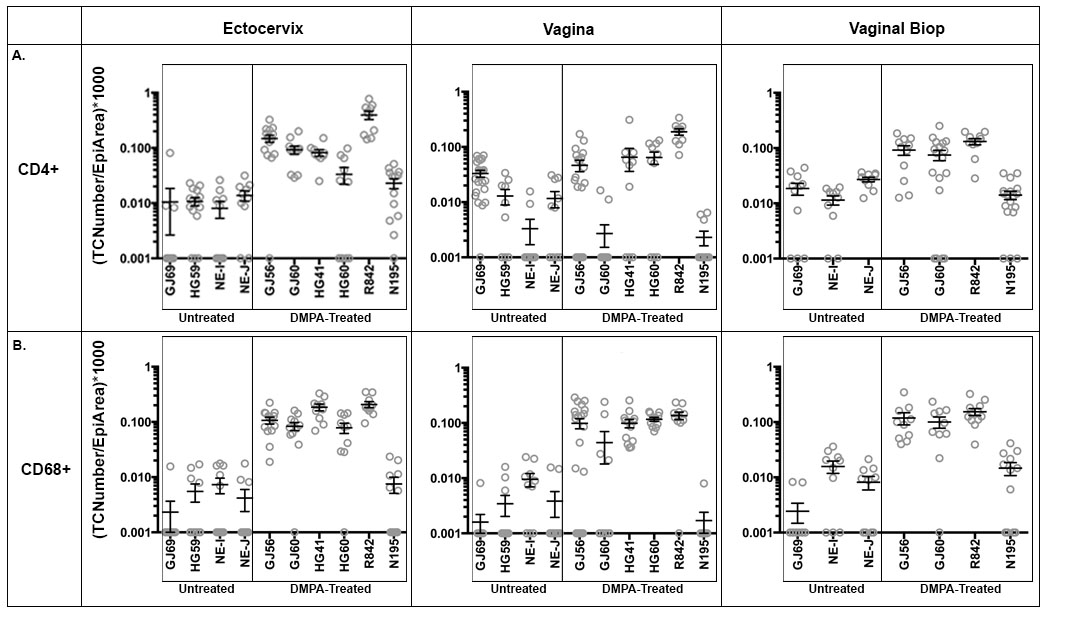

Supplement: S1 Fig — DMPA refers to those animals that were pre-treated with intramuscular injections of 30 mg Depo-provera 4–5 weeks (28–33 days) prior to sacrifice. TCNumber/EpiArea refers to the number of target cells divided by the area of the epithelium analyzed. Each data point represents the mean cell density from a 40x panel image. Each animal had 10 panel images, 1 panel per random section, taken per tissue type from multiple blocks when available. Error bars represent SEM. (a). Analysis of CD4+ T-cell density in untreated (n = 4) and DMPA-treated rhesus macaques (n = 6), comparing terminal tissue collections (Ectocervix and Vagina) and vaginal tissue biopsies. (b). Analysis of CD68+ T-cell density in untreated (n = 4) and DMPA-treated rhesus macaques (n = 6), comparing terminal tissue collections (Ectocervix and Vagina) and vaginal tissue biopsies. (JPG) [file ppat.1005885.s001.jpg]

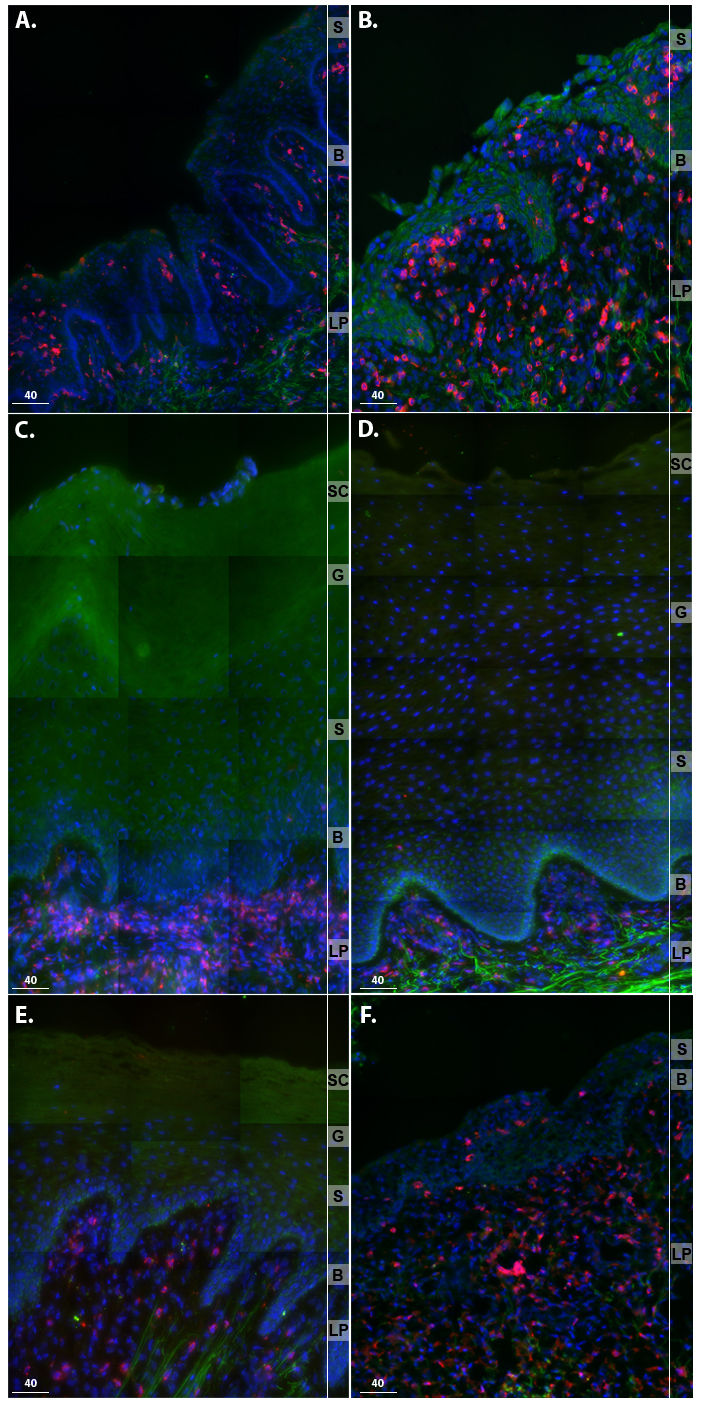

Supplement: S2 Fig — Fluorescent deconvolution images (40x) of various macaque samples. CD4+ T-cells (red), DAPI (blue), tissue background (green). Size bars are 40μm. Tissue layers are labeled as follows: SC = stratum corneum, G = granulosum, S = spinosum, and B = basal layer, LP = lamina propria. (a). Vaginal epithelium from a DMPA-treated rhesus macaque (30mg). (b). Vaginal epithelium from a DMPA-treated infected pigtail macaque (30mg). (c). Vaginal epithelium from an untreated rhesus macaque. (d). Vaginal epithelium from the midcycle menstrual cycle phase in an infected pigtail macaque. (e). Vaginal epithelium from the follicular menstrual cycle phase in an infected pigtail macaque. (f). Vaginal epithelium from the luteal menstrual cycle phase in an infected pigtail macaque. (JPG) [file ppat.1005885.s002.jpg]

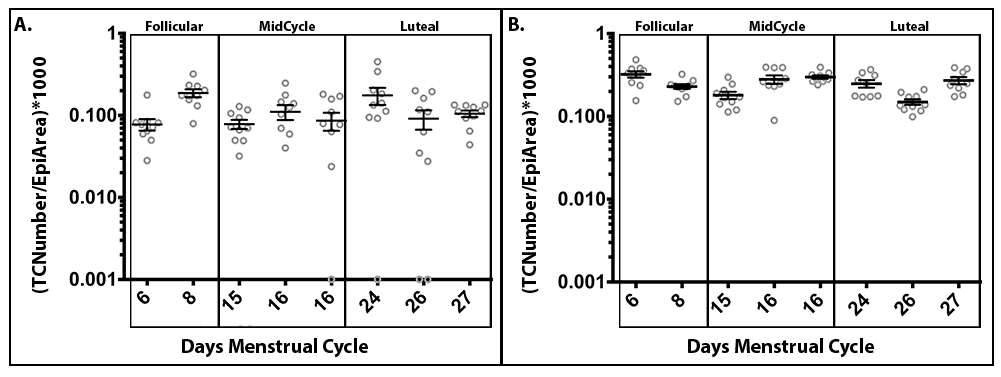

Supplement: S3 Fig — Menstrual cycle phases were designated as follicular (day 1 of menstruation until day 14), midcycle (days 14–16), or luteal (days 17 to day prior to menstruation). TCNumber/EpiArea refers to the number of target cells divided by the area of the epithelium analyzed. Each data point represents the mean cell density from a 40x panel image. Each animal had 10 panel images, 1 panel per random section, taken per tissue type from multiple blocks when available. Error bars represent SEM. (a). Analysis of CD4+ T-cell density in the simple columnar epithelium of infected pigtail macaques by phase of the menstrual cycle. (b). Analysis of CD68+ macrophage density in the simple columnar epithelium of infected pigtail macaques by phase of the menstrual cycle. (JPG) [file ppat.1005885.s003.jpg]

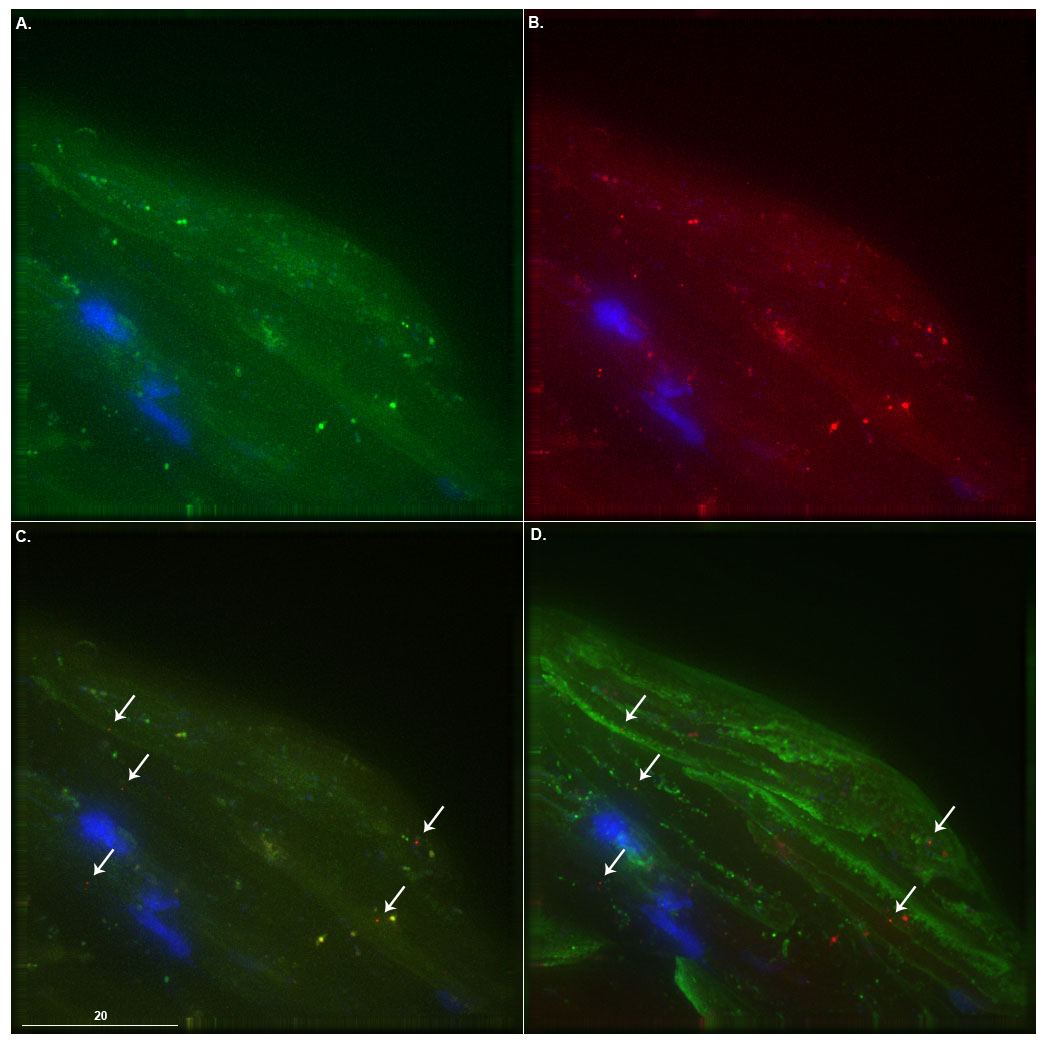

Supplement: S4 Fig — Fluorescent deconvolution images (100x) of macaque vagina. Size bars are 20μm. (a) GFP signal before photoactivation, illustrating tissue autofluorescence (green) and DAPI (blue). (b) GFP signal after photoactivation (pseudo-colored red) and DAPI (blue). (c) An overlay of GFP signal before (A) and after photoactivation (B). Identified virions are shown (red, white arrows), pre-photoactivation (green) and DAPI (blue). (d) Identified virions are shown (red), WGA (green), and DAPI (blue). (JPG) [file ppat.1005885.s004.jpg]

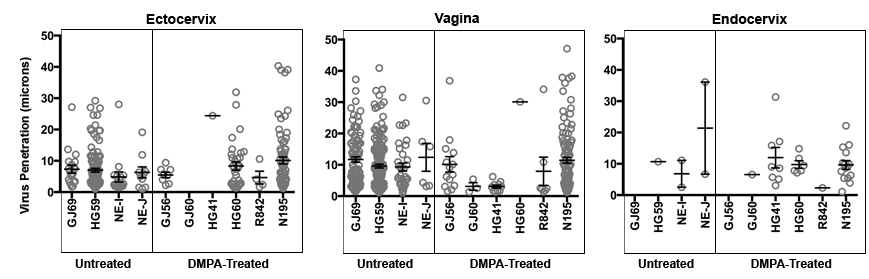

Supplement: S5 Fig — DMPA refers to those animals that were pre-treated with intramuscular injections of 30 mg Depo-provera 4–5 weeks (28–33 days) prior to sacrifice. Analysis of PA-GFP HIV-1 virus penetration in untreated (n = 4) and DMPA-treated rhesus macaques (n = 6), comparing terminal tissue collections (ectocervix and vagina) and vaginal tissue biopsies. Each data point displayed represents individual penetrating virions. Each animal had ~20 100x images for each available block of each tissue type. Error bars represent SEM. (JPG) [file ppat.1005885.s005.jpg]
